# Nanoparticle composite TPNT1 is effective against SARS-CoV-2 and influenza viruses

**DOI:** 10.1038/s41598-021-87254-3

**Published:** 2021-04-22

**Authors:** Sui-Yuan Chang, Kuo-Yen Huang, Tai-Ling Chao, Han-Chieh Kao, Yu-Hao Pang, Lin Lu, Chun-Lun Chiu, Hsin-Chang Huang, Ting-Jen Rachel Cheng, Jim-Min Fang, Pan-Chyr Yang

**Affiliations:** 1 Department of Clinical Laboratory Sciences and Medical Biotechnology, National Taiwan University College of Medicine, No. 1, Sec. 1, Ren-Ai Rd., Taipei, 10002 Taiwan; 2 Department of Laboratory Medicine, National Taiwan University Hospital, Chung-Shan South Rd., No. 7, Taipei, 10002 Taiwan; 3 Department and Graduate Institute of Microbiology and Immunology, National Defense Medical Center, Taipei, 11490 Taiwan; 4 Tripod Nano Technology, No. 171, Sec. 1, Mei Shi Rd., Yang Mei District, Taoyuan, 32656 Taiwan; 5 The Genomics Research Center, Academia Sinica, No. 128, Sec. 2, Academia Rd., Taipei, 11529 Taiwan; 6 Department of Chemistry, National Taiwan University, No. 1, Sec. 4, Roosevelt Rd., Taipei, 10607 Taiwan; 7 Department of Internal Medicine, National Taiwan University Hospital and National Taiwan University College of Medicine, No. 7, Chung-Shan South Rd., Taipei, 10002 Taiwan; 8 Institute of Biomedical Sciences, Academia Sinica, No. 128, Sec. 2, Academia Rd., Taipei, 11529 Taiwan

**Keywords:** Nanoparticles, Antiviral agents

## Abstract

A metal nanoparticle composite, namely TPNT1, which contains Au-NP (1 ppm), Ag-NP (5 ppm), ZnO-NP (60 ppm) and ClO_2_ (42.5 ppm) in aqueous solution was prepared and characterized by spectroscopy, transmission electron microscopy, dynamic light scattering analysis and potentiometric titration. Based on the in vitro cell-based assay, TPNT1 inhibited six major clades of severe acute respiratory syndrome coronavirus 2 (SARS-CoV-2) with effective concentration within the range to be used as food additives. TPNT1 was shown to block viral entry by inhibiting the binding of SARS-CoV-2 spike proteins to the angiotensin-converting enzyme 2 (ACE2) receptor and to interfere with the syncytium formation. In addition, TPNT1 also effectively reduced the cytopathic effects induced by human (H1N1) and avian (H5N1) influenza viruses, including the wild-type and oseltamivir-resistant virus isolates. Together with previously demonstrated efficacy as antimicrobials, TPNT1 can block viral entry and inhibit or prevent viral infection to provide prophylactic effects against both SARS-CoV-2 and opportunistic infections.

## Introduction

The ongoing coronavirus disease 2019 (COVID-19) pandemic has imposed on tremendous threat to humans and global socioeconomics. The causative agent of COVID-19, SARS-CoV-2, has caused more than 42,512,186 infections and 1,147,301 deaths after spreading into 218 countries^1^. With the efficient spreading of SARS-CoV-2, it is becoming more difficult to contain the virus transmission. Many researchers have exerted great efforts to develop effective vaccines and antiviral drugs against SARS-CoV-2. One strategy is to re-purpose the existing broad-spectrum antiviral agents for COVID-19 therapeutics^2–4^. For example, remdesivir, favipiravir, lopinavir and hydroxychloroquine at micromolar concentrations have been reported to inhibit SARS-CoV-2 based on the in vitro assays. Remdesivir is even recently applied in compassionate use for patients with severe COVID-19 infection^5^.

In contrast to the small molecules, we consider using metal nanoparticles as the prophylactic of COVID-19 infection. Metal nanoparticles may interact with virus surface glycoproteins and thus interfere with viral attachment and entry into host cells^6^. Metal nanoparticles may also exert antiviral activity by interaction with viral genomes^7^. Silver nanoparticle (Ag-NP), zinc oxide nanoparticle (ZnO-NP) and gold nanoparticle (Au-NP) are well-known antimicrobial agents against many kinds of bacteria, fungi and viruses^7–10^. Silver nanoparticles are antiviral agents against various types of viruses including hepatitis B virus (HBV), herpes simplex virus type 1 (HSV-1), human immunodeficiency virus type 1 (HIV-1) and influenza virus, in addition to the antimicrobial activity against both Gram-positive and Gram-negative bacteria such as *Bacillus subtilis*, *Escherichia coli, Pseudomonas aeruginosa*, *Vibrio cholera* and methicillin-resistant *Staphylococcus aureus* (MRSA). A possible antimicrobial mechanism of Ag-NP is attributable to the suppression of respiratory enzymes and interference with DNA functions by the released Ag^+^ ions^9^. ZnO-NP can inhibit the growth and biofilm formation of MRSA, *Streptococcus pneumoniae*, *Klebsiella pneumoniae* and *Pseudomonas aeruginosa*. On exposure to UV-radiation, ZnO-NP further acts as a photocatalyst for generation of reactive oxygen species to damage viruses and bacteria^11^. A recent review highlights the role of Zn^2+^ ion in antiviral activity including inhibition of SARS-CoV RNA polymerase and angiotensin-converting enzyme 2 (ACE2), which is a receptor for SARS-CoV-2 entry into host cells^12^. Au-NP, generally used as a carrier in photodynamic therapy and biomedical applications, also shows potential use in suppressing HIV-1 and *Mycobacterium tuberculosis*^13, 14^.

In this study, we aimed to determine the antiviral activity of a metal nanoparticle composite, TPNT1, against SARS-CoV-2 and another respiratory pathogen, influenza virus, which also exhibit the potential to trigger a global outbreak in humans.

## Results

**Figure 1:**
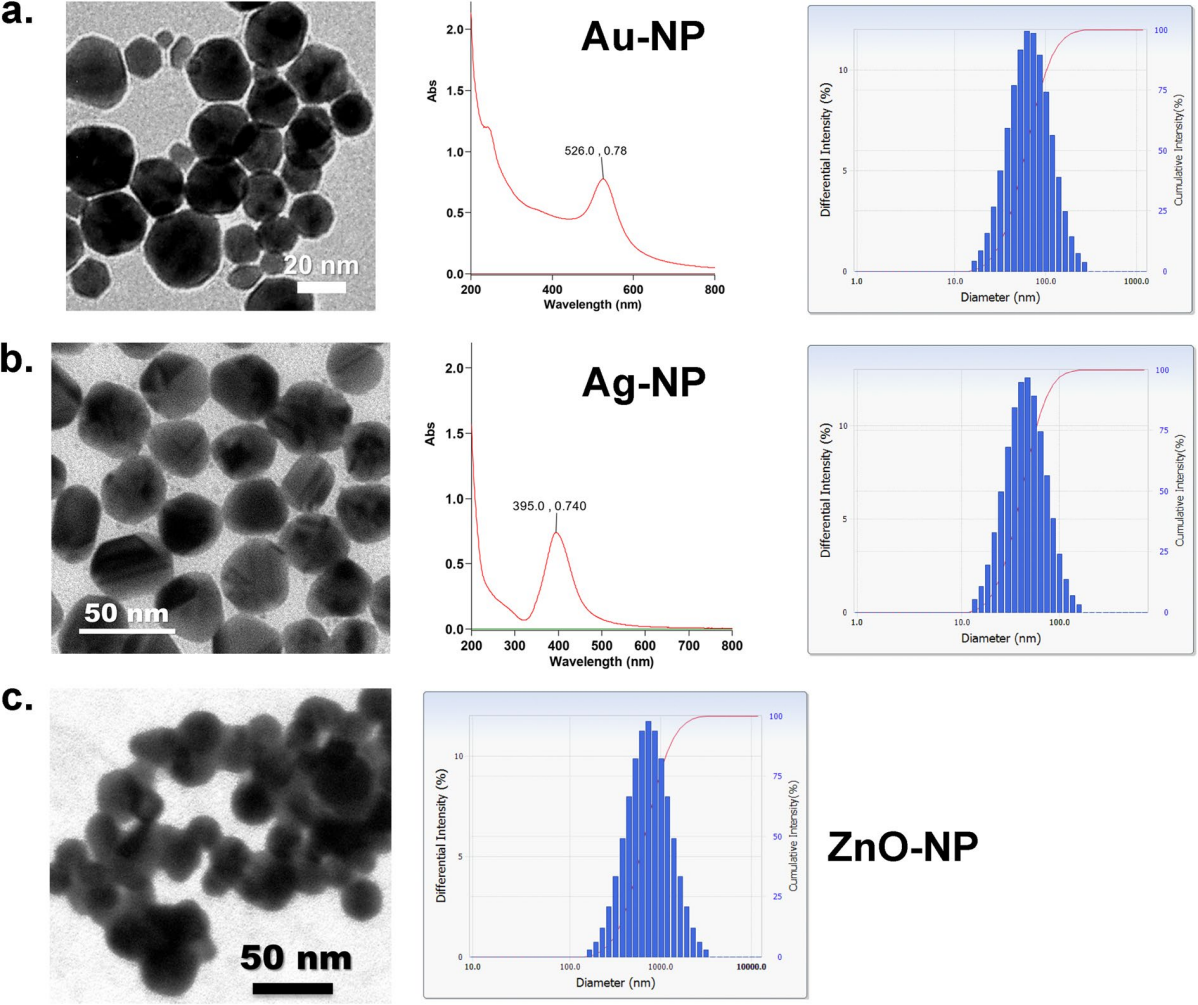
Transmission electron microscopy (TEM) images, UV–vis spectra and dynamic light scattering (DLS) analyses of metal nanoparticles. (**a**) Au-NP, (**b**) Ag-NP, and (**c**) ZnO-NP.

In this study, we formulated a metal nanoparticle composite, TPNT1 as the stock solution, which contains Au-NP (1 ppm), Ag-NP (5 ppm), ZnO-NP (60 ppm) and ClO_2_ (42.5 ppm) in aqueous solution with a positive zeta potential of + 32.81 mV. The individual metal nanoparticles were synthesized according to our patented method^15^. In brief, a metal aqueous solution (HAuCl_4_, AgNO_3_ or ZnCl_2_) was reduced by heating with citric acid at 150 °C for 12 min, and then dispersed in an appropriate medium to obtain the colloidal metal nanoparticles. The physicochemical properties of the synthesized nanoparticles were fully characterized by ultraviolet–visible spectroscopy, infrared spectroscopy, inductively coupled plasma atomic emission spectroscopy, transmission electron microscopy (TEM), dynamic light scattering (DLS) analysis and potentiometric titration (Fig. 1). According to the TEM imaging, Au-NP, Ag-NP and ZnO-NP are in spherical shape with 20–40, 10–40 and 25–35 nm diameters, respectively. The average sizes of colloidal Au-NP, Ag-NP and ZnO-NP are 78.1, 50.4 and 619.1 nm as shown individually by the DLS analysis. A colloidal solution of nanoparticle composite, namely TPNT1, containing 1 ppm Au-NP, 5 ppm Ag-NP, 60 ppm ZnO-NP and 42.5 ppm chlorine dioxide (ClO_2_) was prepared by well mixing of the above-described materials.

**Figure 2:**
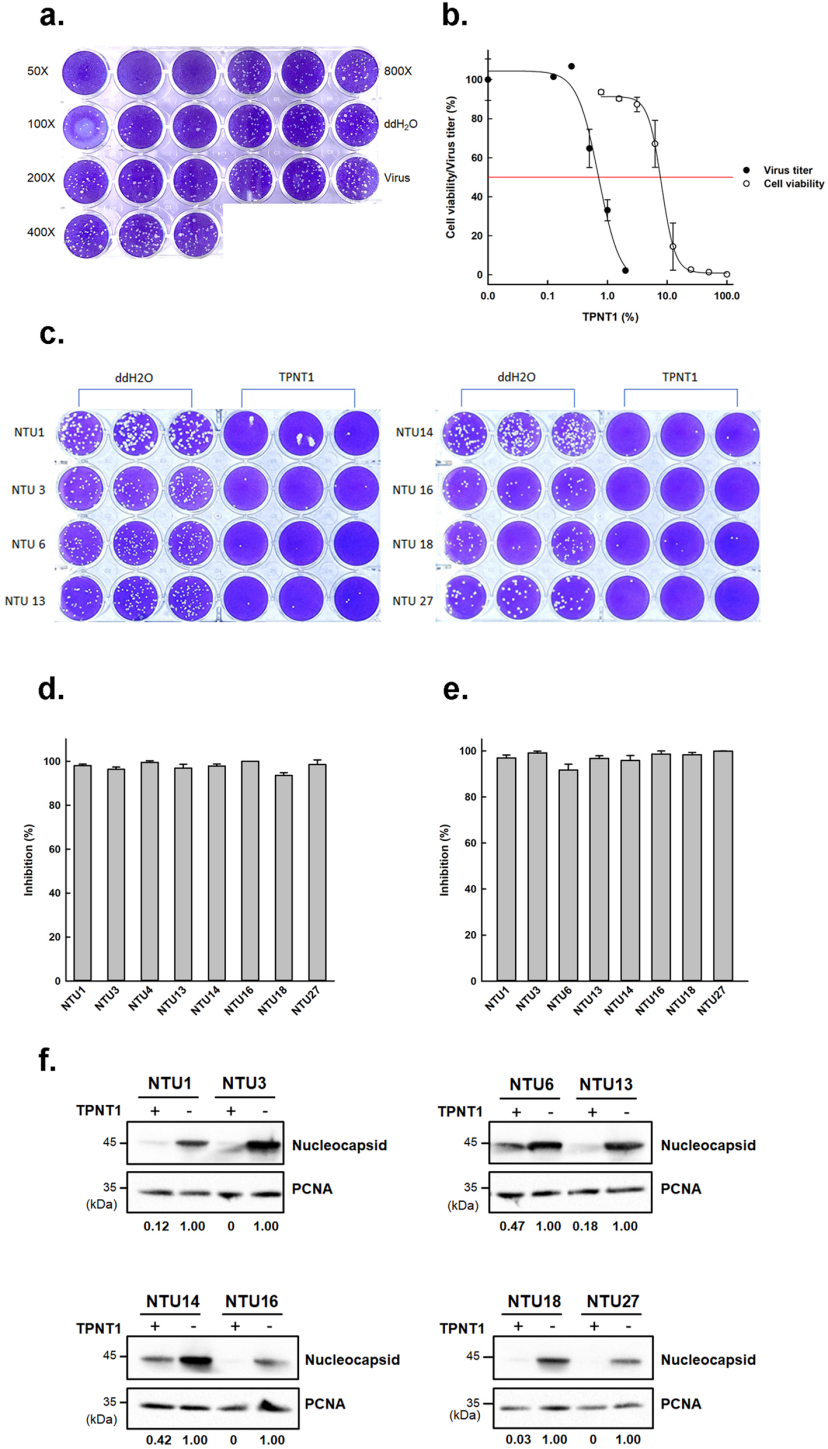
The antiviral activities of TPNT1 against SARS-CoV-2 in vitro. (**a**) Inhibition of plaque formation in the presence of serially diluted TPNT1. (**b**)The half maximal effective concentration (EC_50_) and cell toxicity of TPNT1 for Vero E6 cells was determined by plaque reduction assay and ACP assay, respectively. (**c**) Plaque reduction assay of TPNT1(1:50 dilution) against various SARS-CoV-2 strains. (**d**) The percentage of inhibition by plaque reduction assay in (**c**). (**e**) Inhibition of virus titers in the supernatants of SARS-CoV-2 infected H1975-ACE2 cells by TPNT1. (**f**) Inhibition of viral nucleocapsid (NP) protein expression in TPNT-1 treated SARS-CoV-2 infected H1975-ACE2 cells. The ratio of NP to the internal loading control, proliferating cell nuclear antigen (PCNA), was shown below each blot. At least three independent experiments were performed and one representative result was shown. Full-length blots/gels of (**f**) were presented in Supplementary Fig. 1.

The antiviral activity of TPNT1 against SARS-CoV-2 was first examined by plaque reduction assay. Briefly, Vero E6 cells were infected with SARS-CoV-2 (SARS-CoV-2/NTU01/TWN/human/2020) in the presence of various concentrations of TPNT1. As shown in Fig. 2a, an obvious reduction of plaque numbers was observed in the presence of 100-fold diluted TPNT1 containing 0.01 ppm Au-NP, 0.05 ppm Ag-NP, 0.6 ppm ZnO-NP, and 0.425 ppm ClO_2_. The calculated IC_50_ for TPNT1 is 143 ± 15.5-fold dilution (Fig. 2b). The cell toxicity of TPNT1 was determined by the acid phosphatase (ACP) assay^16^ and the derived selectivity index (SI; CC_50_/IC_50_) was greater than 10 (Fig. 2b). The ability of TPNT1 to inhibit various SARS-CoV-2 strains was subsequently determined using 7 additional clinical isolates representing 6 major clades of SARS-CoV-2 viruses (Fig. 2c). The virus isolates used were summarized in Table 1. NTU01, NTU03, NTU06, NTU13, NTU14, NTU16, NTU18, and NTU27 represent A, B.1, B.2.2, A3, B.1.1, B.1.5, A.1, and B lineages of SARS-CoV-2, respectively. Among the eight clinical isolates, three (NTU3, 14, and 16) contains the D614G mutation, which is circulating predominantly worldwide since March, 2020, and has been reported to exhibit increased viral infectivity^17^. As shown in Fig. 2d, TPNT1 could inhibit 93.5–100% of plaque formation by these SARS-CoV-2 strains. The ability of TPNT1 to inhibit virus replication was also examined using yield-reduction assay in a human lung adenocarcinoma cell line H1975 transduced with ACE2 (H1975-ACE2). The viral titers in the culture supernatants was determined by plaque assay. A significant inhibition of infectious virus titers in the culture supernatants was observed for all clinical isolates, including the D614G variants, in the presence of TPNT1 by plaque assay (Fig. 2e). TPNT1 can also inhibit the viral nucleoprotein (NP) expression in H1975-ACE2 cells infected by these SARS-CoV-2 strains (Fig. 2f).

**Table 1:** Genetic characteristics of SARS-CoV-2 isolates used in this study.

Virus name	ORF1a						ORF1a				S		NS3		NS8	N				GISAID	Lineage	Accession ID
	NSP1		NSP2	NSP3	NSP6	NSP8	NSP12	NSP13		NSP14												
	D75	G82	H237	P153	L37	M129	P323	P504	Y541	F233	D614	R756	Q57	G251	L84	R203	G204	A205	T393			
hCoV-19/Taiwan/NTU01/2020	–	–	–	–	–	–	–	–	–	–	–	–	–	–	S	–	–	–	–	S	A	EPI_ISL_408489
hCoV-19/Taiwan/NTU03/2020	–	–	–	–	–	–	L	–	–	–	G	–	H	–	–	–	–	–	–	GH	B.1.255	EPI_ISL_413592
hCoV-19/Taiwan/NTU06/2020	–	–	R	–	F	–	–	–	–	–	–	–	–	V	–	–	–	–	–	V	B.39	EPI_ISL_422409
hCoV-19/Taiwan/NTU013/2020	E	–	–	L	–	I	–	–	–	L	–	–	–	–	S	–	–	G	I	S	A.3	EPI_ISL_422415
hCoV-19/Taiwan/NTU014/2020	–	–	–	–	–	–	L	–	–	–	G	L	–	–	–	K	R	–	–	GR	B1.1.119	EPI_ISL_422416
hCoV-19/Taiwan/NTU016/2020^a^	–	D	–	–	–	–	L	–	–	–	G	–	–	–	–	–	–	–	–	G	B.1	EPI_ISL_42218
hCoV-19/Taiwan/NTU018/2020^a^	–	–	–	–	–	–	–	L	C	–	–	–	–	–	S	–	–	–	–	S	A.1	EPI_ISL_447615
hCoV-19/Taiwan/NTU027/2020^a^	–	–	–	–	–	–	–	–	–	–	–	–	–	–	–	–	–	–	–	L	B	EPI_ISL_447621

**Figure 3:**
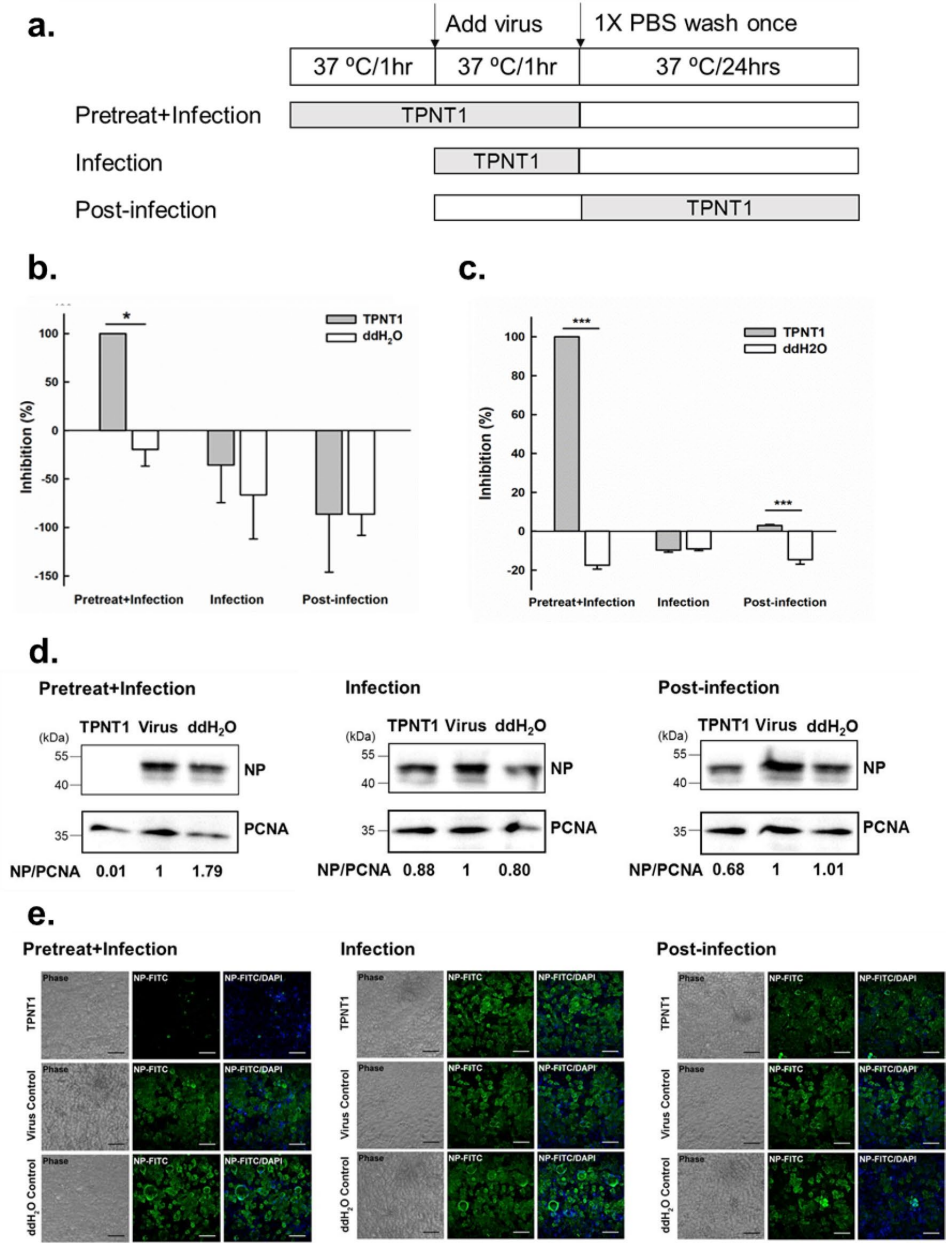
Inhibition of SARS-CoV-2 entry by TPNT1. (**a**) Schematic illustration of the (pretreat + infection), infection-only, and post-infection experiments delineates the stage where TPNT1 was added to the viruses or the cells; (**b**) Quantification of the viral RNA in the supernatants of SARS-CoV-2 infected Vero E6 cells; (**c**) qRT-PCR detection of viral RNA in the SARS-CoV-2 infected Vero E6 cells ; (**d**) Western blot of the viral nucleocapsid (NP) protein; and (**e**) Immunofluorescence assay for the detection of SARS-CoV-2 NP probed with a rabbit monoclonal antibody. Scale bars: 100 µm. At least three independent experiments were performed and one representative result was shown. *P* < 0.05*; *P* < 0.001*** Full-length blots/gels of (**d**) were presented in Supplementary Fig. 2.

Yield reduction assay was subsequently performed to determine the stage where TPNT1 might exert its effects by either pretreating the virus with TPNT1 (pretreat + infection), adding TPNT1 during (infection) or after virus infection (post-infection). The schematic illustration for experimental design was shown in Fig. 3a. The culture supernatants and cell lysates were harvested for subsequent analysis. The virus copy number in the culture supernatant was determined by quantitative real-time RT-PCR (qRT-PCR) (Fig. 3b). The replication of virus in the infected cells was determined by quantification of intracellular viral mRNA and viral nucleocapsid protein (NP) expression using the qRT-PCR of oligo-dT amplified cDNA (Fig. 3c), western blot analysis (Fig. 3d), and immunofluorescence assay (Fig. 3e), respectively. Based on the experimental results, pre-incubation of diluted TPNT1 with SARS-CoV-2 is required for efficient inhibition of SARS-CoV-2 replication. TPNT1 functioned at a stage before virus infection since the virus titers in the supernatants reduced significantly when TPNT1 was preincubated with virus before infection (Fig. 3b). Corresponding reduction of intracellular viral RNA and viral NP protein expression was only observed in cells infected with TPNT1-pretreated viruses (Fig. 3c–e).

**Figure 4:**
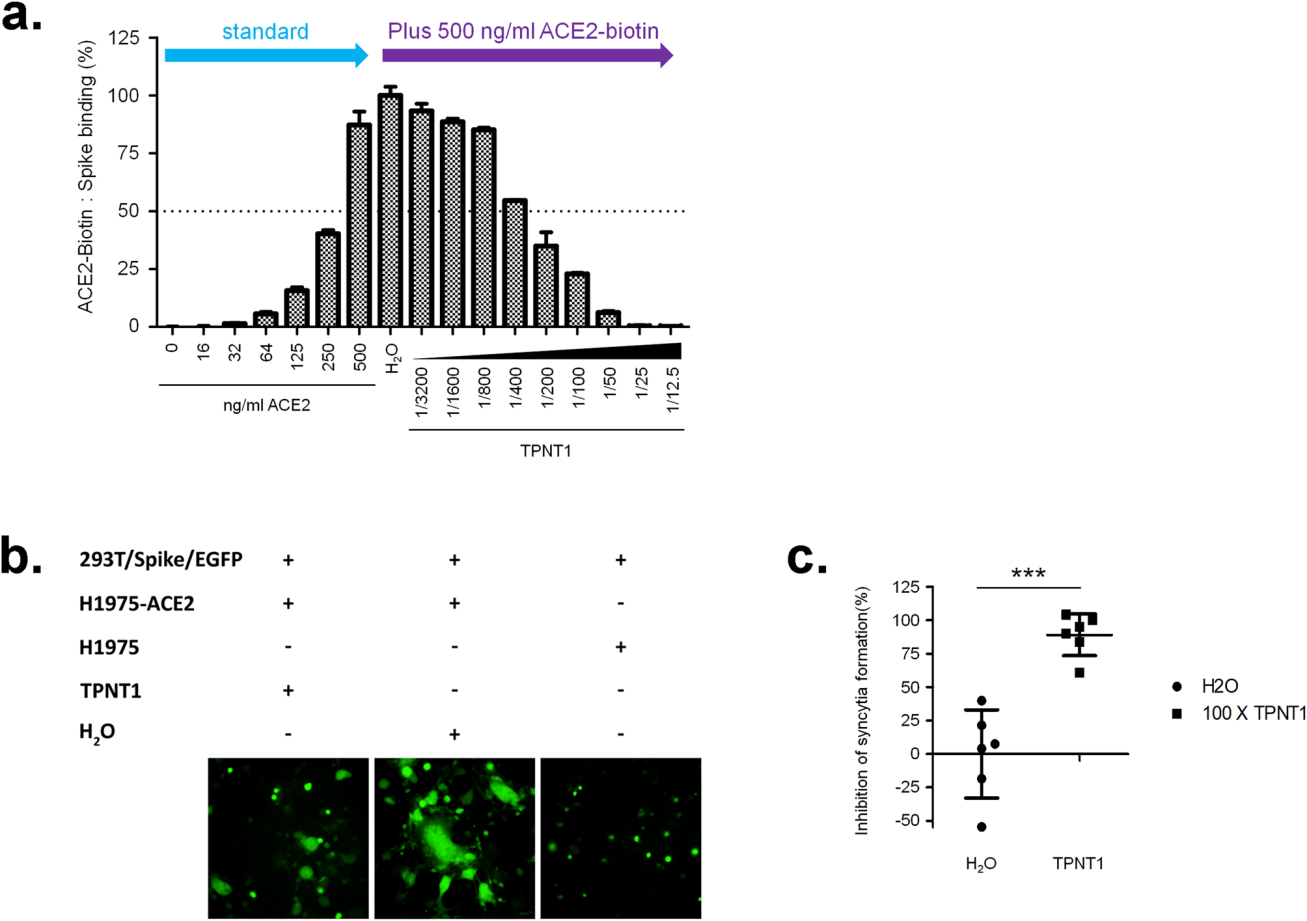
Inhibition of SARS-CoV-2 entry by TPNT1. (**a**) TPNT1 blocks binding of spike proteins to ACE2-Fc-Biotin; (**b** & **c**) TPNT1 inhibited syncytium formation between 293 T/Spike/EGFP and H1975-ACE2 cells. At least three independent experiments were performed and one representative result was shown. *P* < 0.001***.

The ability of TPNT1 to inhibit binding of SARS-CoV-2 spike protein to ACE2 receptor was also confirmed by the ELISA assay using ACE2-Fc-Biotin and spike protein. As shown in Fig. 4a, TPNT1 blocked spike protein from binding to ACE2-Fc-Biotin in a dose-dependent manner. Since syncytium formation is a step critical for virus entry after receptor binding, we also examined the ability of TPNT1 to inhibit syncytium formation. As shown in Fig. 4b, syncytium formation between 293 T/Spike/EGFP and H1975-ACE2 cells was inhibited significantly in the presence of TPNT1. The inhibition of syncytia formation was calculated by counting the 293 T/Spike/EGFP cells fused or unfused with H1975-ACE2 cells under an inverted fluorescence microscope. A significant reduction of syncytium formation was observed in the presence of TPNT1 as compared to the solvent (H_2_O) control (Fig. 4c).

**Figure 5:**
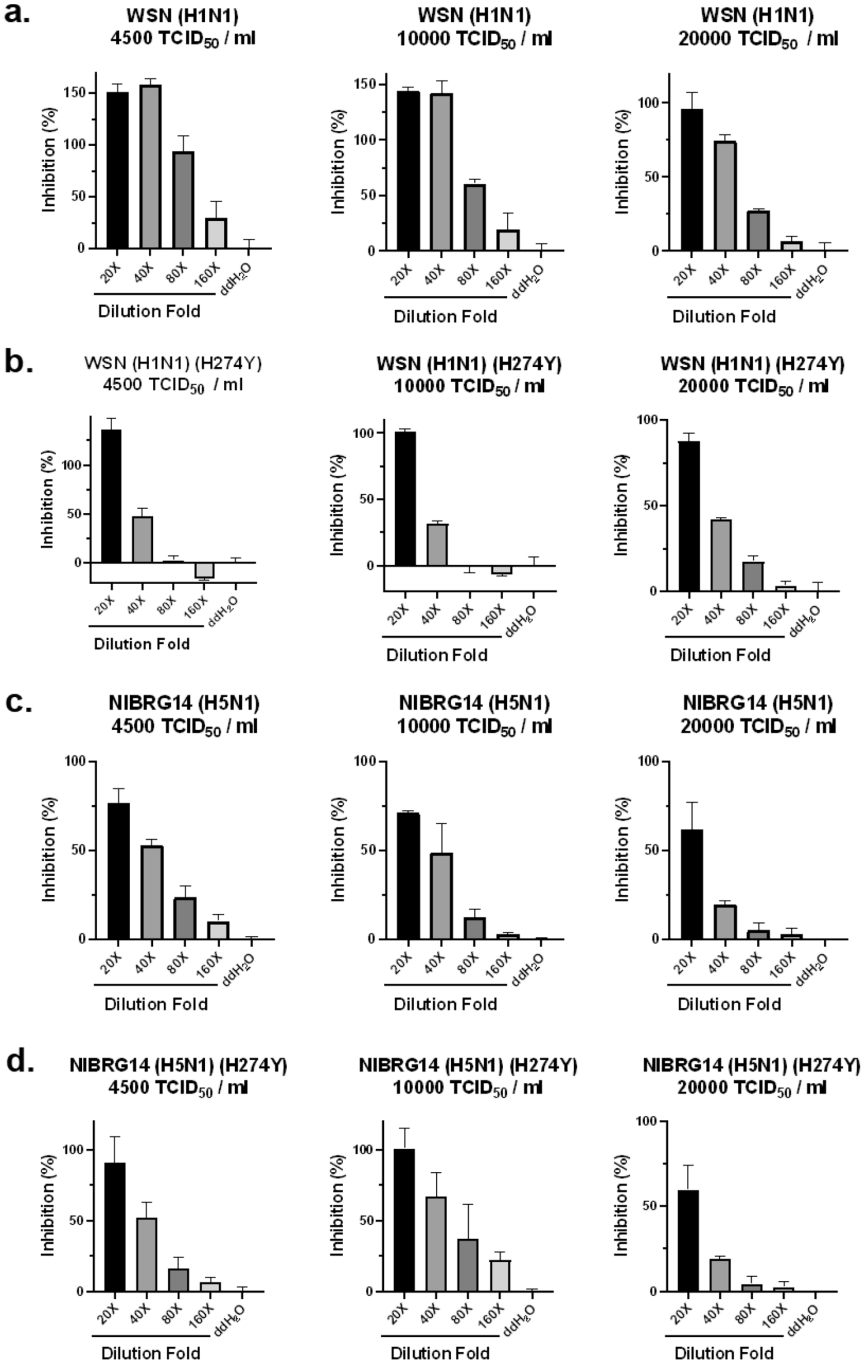
The antiviral activities of TPNT1 against influenza viruses in vitro. MDCK cells were incubated with (**a**) WSN (H1N1), (**b**) oseltamivir-resistant WSN (H1N1)(H274Y), (**c**) NIBRG14 (H5N1), and (**d**) oseltamivir-resistant NIBRG14 (H5N1)(H274Y) at various viral inputs in the presence of TPNT1. After 48 h at 35 °C, the virus-induced cytotoxicity was monitored. The cells incubated with virus only were defined as 0% while the cells with no virus inoculation were defined as 100% inhibition.

In addition to SARS-CoV-2, we also tested the antiviral activities of TPNT1 against influenza virus infection in cell-based assays. Although there are currently four FDA-approved influenza antiviral drugs, peramivir, zanamivir, oseltamivir phosphate, and baloxavir marboxil, drug-resistant viruses have emerged and new therapeutics targeting drug-resistant viruses are needed^18–21^. In addition, the threat from avian influenza A virus to cause pandemics in human population cannot be ignored ^22^. Therefore, the antiviral activity of TPNT1 against seasonal influenza A (H1N1) and avian influenza A virus (H5N1) was determined by measuring the cytopathic effects (CPE) induced by virus infection. The inhibitory effects of TPNT1 was examined using three different virus inputs, 4,500, 10,000, and 20,000 TCID_50_/mL. As shown in Fig. 5a and c, TPNT1 effectively relieved the cells from virus-induced cytopathic effects, and a very modest reduced inhibition by TPNT1 was observed when the cells were infected with extremely high viral loads (20,000 TCID_50_/mL). The activities of TPNT1 against oseltamivir-resistant influenza viruses were also examined (Fig. 5b and d). Similar inhibitory effects against wild-type and oseltamivir-resistant influenza viruses were observed, although a slightly reduced inhibition against oseltamivir-resistant H1N1 viruses was noticed.

## Discussion

The person-to-person contact through respiratory droplets generated by sneezing and coughing, or contaminated fomites from the infected individuals has been shown to be the major transmission route for SARS-CoV-2. Besides efficient use of protective personal equipment, such as masks, as well as keeping social distance, implementation of a more active prevention strategy is required to contain the SARS-CoV-2 pandemic. In this study, we demonstrated the efficiency of a metal nanoparticle composite, TPNT1, as the prophylactic of COVID-19 infection. In addition, the anti-influenza activity of TPNT1 was also examined because influenza virus, known to infect millions of people annually and causing severe diseases, especially in the elderly^23^, shares the similar transmission routes, clinical presentations and seasonal coincidence with SARS-CoV-2. Based on the in vitro cell-based assay, TPNT1 was shown to efficiently inhibit SARS-CoV-2 as well as human (H1N1) and avian (H5N1) influenza viruses, likely through blocking of the viral entry. Receptor binding represents a critical step for viral entry and subsequent virus replication in host cells. Interactions between the SARS-CoV-2 spike protein and the ACE2 receptor^24, 25^, and the hemagglutinin (HA) of avian and human influenza viruses with the 2,3-linked or 2,6-linked Neu5Ac receptor on host cells^26^ have been shown to be essential for virus infection. We demonstrated that TPNT1 can efficiently abrogate the binding of SARS-CoV-2 spike protein to the ACE2 receptor on host cells, and to prevent the formation of syncytial cells. These data supports that TPNT1 can block viral entry and subsequently prevent viral infection to provide prophylactic effects against both SARS-CoV-2 and influenza viruses.

Metal nanoparticles with large surface area can conjugate modifiers to attain multivalent effect on targeting viruses. For example, the Au-NPs conjugated with multivalent sialic-acid-terminated glycerol dendrons can tightly bind influenza hemagglutinin, and thus interfere with the attachment of virus to host cell^27^. However, preparation of organic modifiers and encapsulation of nanoparticles usually require tremendous synthetic effort. In comparison, we used “naked” Au-NPs without bioconjugation, along with Ag-NPs, ZnO-NPs and ClO_2_, as an effective composite agent to inhibit the binding of viruses to host cell. The SARS-CoV-2 and influenza virions, having average diameters of 80–120 nm, are in the size range of nanoparticles^28, 29^. As TPNT1 is most effective by pre-incubation with viruses, one possible mechanism for the antiviral activity of TPNT1 may be attributable to bindings of virus surface glycoproteins with the metal nanoparticles, and thus preventing the virions from attachment to host cells^27, 30–33^. Indeed, our experiments (Fig. 4) support that TPNT1 can block viral entry by inhibiting the binding of SARS-CoV-2 spike proteins to ACE2 receptor and to interfere with the syncytium formation. It has been suggested that nanoparticles can diminish virus entry by direct interaction with the sulfur-bearing residues on the viral surface^34^. Based on the sequence analysis, SARS-CoV-2 spike has 40 cysteine residues while influenza hemagglutinin has 15 cysteine residues. Moreover, TPNT1 has positive surface charge (+ 32.81 mV) that may also enhance the binding with virions^35^. Inclusion of ClO_2_ as an oxidizing agent in TPNT1 nanometal composite is beneficial for virus inhibition. A previous report indicates that magnesium oxide nanoparticle (MgO-NP) impregnated with Cl_2_ exhibits higher bactericidal activity than free Cl_2_ or MgO-NP itself^36^. Another study shows that the combination of single-wall carbon nanotubes and NaOCl (or H_2_O_2_) also displays a synergistic sporicidal effect^37^. We assume that the ClO_2_ component in TPNT1 may render oxidative damage of virus surface, and thus provide enhanced effect for viral inhibition by the metal nanoparticles.

Although metal nanoparticles have been demonstrated to have a wide range of biomedical applications^38^, the toxicity is an issue of concern. Most metal nanoparticles have beneficial and adverse dual effects. The degree of toxicity varies by the type, shape, size, purity, concentration, administration method and exposure time of metal nanoparticles. The current available data from many research teams are insufficient, and some are even contradictory, to determine the adverse effects of metal nanoparticles on human health^39–48^. As the information of toxicological studies including bio-distribution and metabolism of nanosized particles are insufficient, the safe doses of Au-NP, Ag-NP and ZnO-NP for humans are not yet established by European Food Safety Authority (EFSA) or US Environmental Protection Agency (EPA). In this study, all the Au-NP, Ag-NP and ZnO-NP in TPNT1 are prepared in spherical form, and spherical metal nanoparticles are known to be non-toxic or less toxic than that in other shapes^49, 50^. Gold compounds have been used in medicine for decades and most studies on animal models support that AuNPs do not cause appreciable toxicity^51^. The previous studies showed that 0.1–2.0 mg/kg was a safe dose of Ag-NP for oral administration to mice and rats^52^. The recommended daily intake of zinc is 8–11 mg for adults, and ZnO-NP at low dose (100 mg/kg bwt/day) is beneficial^53^. The amounts of Au (< 0.01 ppm), Ag (< 0.05 ppm) and ZnO (< 0.6 ppm) in the 50% effective dose of TPNT1 tests are considered within the range of concentration to be used as food additives. The content of ClO_2_ (< 0.425 ppm) is also within the safety concentration in drinking water^54, 55^.

In summary, we have shown that TPNT1 could effectively inhibit different strains of SARS-CoV-2 as well as human H1N1 and avian H5N1 influenza viruses, including the oseltamivir-resistant H274Y strains. Although we do not know its detailed anti-entry mechanism, TPNT1 indeed showed broad spectrum of antiviral activities against various SARS-CoV-2 strains and human/avian influenza viruses. Considering its prophylactic application, inorganic metal nanoparticles-based TPNT1 will have a relatively lower tendency to induce resistant viruses compared with therapeutic organic drugs. We believe TPNT1 can provide prophylactic effects against SARS-CoV-2 and opportunistic infections which are frequently observed in patients suffering SARS-CoV-2 infection by oral gargling, nasal spray, nebulized inhalation or even systemic use after an appropriate clinical trial.

## Materials and methods

### General

All the reagents were reagent grade and used as purchase without further purification. Tetrachloroauric acid (HAuCl_4_, 0.2 M aqueous solution) and zinc powder were purchased from Acros Organics (New Jersey, USA). Silver nitrate (AgNO_3_, 0.1 M aqueous solution) was purchased from Merck & Co. (New Jersey, USA). Ultra-pure water was purchased from Hao Feng Biotech Co. (Taipei, Taiwan). All cell culture reagents were obtained from Invitrogen Inc. (Carlsbad, CA, USA).

The transformations in the surface plasmonic resonance of Au-NP and Ag-NP were measured on an Agilent Technologies Cary60 ultraviolet–visible (UV–vis) spectrophotometer operating at a resolution of 2 nm. Infrared (IR) spectra were recorded on an Agilent Technologies Cary630 FT-IR spectrometer. Transmission electron microscopy (TEM) was performed on an FEI Tecnai G2 F-20 S-TWIN operating at an accelerating voltage of 200 kV. Particle size distribution and zeta potential were measured on an Otsuka ELSZ-2000ZS dynamic light scattering (DLS) detector. The content of metal ions was determined by inductively coupled plasma optical emission spectrometry (ICP-OES) on an Agilent 5100 instrument. All the analyses were carried out with triplicate measurements for a single sample.

### Synthesis and characterization of nanoparticles^15^

Tetrachloroauric acid (2.25 mL of 0.2 M aqueous solution, 0.45 mmol) and citric acid (360 mg, 1.87 mmol) were added via an inlet port into a double-necked flat-bottomed 100 mL reaction flask and were mixed to form a mixture solution. Subsequently, the flat-bottomed flask was placed on a hot plate and heated at 130 ℃ for 20 min to perform a reduction reaction, which was monitored by the IR spectrometer. The reduction reaction produced a composition containing gold nanoparticles and HCl gas. At the same time, HCl gas was guided through the recovery port attached to the flat-bottomed flask and was trapped with 10 mL water in an Erlenmeyer flask for collection. Finally, 450 mL of pure water was used as a medium to disperse the gold nanoparticles in the flat-bottomed flask, and said solution was heated at 70 °C. For 10 min to obtain 100 ppm colloidal solution of gold nanoparticles, which showed the TEM size at 20–40 nm, the UV–Vis absorption band at λ_max_ = 526 nm with optical density (OD) = 0.78, and the DLS size at 78.1 nm.

Silver nitrate (15 mL of 0.37 M aqueous solution, 5.5 mmol) and citric acid (4961 mg, 25.59 mmol) were added via an inlet port into a double-necked flat-bottomed 100 mL reaction flask and were mixed to form a mixture solution. Subsequently, the flat-bottomed flask was placed on a hot plat and heated at 150 °C for 35 min to perform a reduction reaction, which was monitored by the IR spectrometer. The reduction reaction produced a composition containing silver nanoparticles and NO_2_ gas. At the same time, NO_2_ gas produced from the reduction reaction was guided through the recovery port attached to the flat-bottomed flask and was trapped with 10 mL water in an Erlenmeyer flask for collection. Finally, an aqueous solution (5100 mL) of citric acid (4026 mg, 20.96 mmol) and NaOH (423 mg, 10.58 mmol) was used as a medium to disperse the silver nanoparticles in the flat-bottomed flask, and said solution was heated at 70 °C for 60 min to obtain 100 ppm colloidal solution of silver nanoparticles, which showed the TEM size at 10–40 nm, the UV–Vis absorption band at λ_max_ = 395 nm with OD = 0.74, and the DLS size at 50.4 nm.

Zinc chloride (8 mL of 2 M aqueous solution, 16 mmol) and citric acid (3608 mg, 18.78 mmol) were added via an inlet port into a double-necked flat-bottomed 100 mL reaction flask and were mixed to form a mixture solution. Subsequently, the flat-bottomed flask was placed on a hot plat and heated at 150 °C for 20 min to perform a reduction reaction, which was monitored by the IR spectrometer. The reduction reaction produced a composition containing zinc nanoparticles and HCl gas. At the same time, HCl gas produced from the reduction reaction was guided through the recovery port attached to the flat-bottomed flask and was trapped with 10 mL water in an Erlenmeyer flask for collection. Finally, an aqueous solution (1050 mL) of citric acid (2688 mg, 13.99 mmol) was used as a medium to disperse the zinc nanoparticles in the flat-bottomed flask, and said solution was heated at 70 °C for 30 min to obtain 250 ppm colloidal solution of zinc nanoparticles, which showed the TEM size at 25–35 nm, and the DLS size at 619.1 nm.

### Preparation of nanoparticle composite TPNT1

The commercially available chlorine dioxide (Taiwan Clean Biochemical Technology Co., Ltd, 1000 ppm) was diluted with double deionized water (ddH_2_O) to a 170 ppm solution. The above-prepared Au-NP (0.5 mL of 100 ppm solution), Ag-NP (2.5 mL of 100 ppm solution), ZnO-NP (12 mL of 250 ppm solution), ClO_2_ (12.5 mL of 170 ppm solution) and ddH_2_O (22.5 mL) were mixed well to form a 50 mL colloidal solution of nanoparticle composite, namely TPNT1, containing 1 ppm Au-NP, 5 ppm Ag-NP, 60 ppm ZnO-NP and 42.5 ppm ClO_2_.

### Cells and SARS-CoV-2

HEK293T, Vero E6, and MDCK cells were purchased from American Type Culture Collection (ATCC) (Manassas, VA, USA) and cultured in Dulbecco's Modified Eagle Medium (DMEM) containing 10% fetal bovine serum (FBS) (Life Technologies). The human lung adenocarcinoma cell line H1975 was generously provided by Prof. Chih-Hsin Yang (Graduate Institute of Oncology, Cancer Research Center, National Taiwan University) and was maintained in RPMI1640 containing 10% FBS. H1975 cells were transduced with lentivirus encoding full-length ACE2 before being used as target cells (H1975-ACE2). The expression of ACE2 in H1975-ACE2 cells was described in more details in another recently submitted manuscript by our group^56^. All adherent cells were cultured at 37 °C in a humidified atmosphere containing 5% CO_2_ and 20% O_2_. Sputum specimens obtained from SARS-CoV-2-infected patients were propagated in Vero E6 cells in DMEM supplemented with 2 μg/mL tosylsulfonyl phenylalanyl chloromethyl ketone (TPCK)-trypsin (Sigma-Aldrich). Culture supernatant was harvested when CPE were seen in more than 70% of cells, and viral titers were determined by a plaque assay. The virus isolates used in the current study were summarized in Table 1. The experimental protocols were approved by the NTUH Research Ethics Committee (202002002RIND by Dr. Jann-Tay Wang), and the informed consent was obtained from all subjects.

### Antiviral activities against SARS-CoV-2

Plaque reduction assay was performed in triplicate in 24-well tissue culture plates. SARS-CoV-2 (100–200 plaque forming unit, pfu/well) was incubated with TPNT1 for 1 h at 37 °C before adding to the cell monolayer for another one hour. Subsequently, virus-TPNT1 mixtures were removed and the cell monolayer was washed once with PBS before covering with media containing 1% methylcellulose for 5–7 days. The cells were fixed with 10% formaldehyde overnight. After removal of overlay media, the cells were stained with 0.5% crystal violet and the plaques were counted. The percentage of inhibition was calculated as [1 − (V_D_/V_C_)] × 100%, where V_D_ and V_C_ refer to the virus titer in the presence and absence of the compound, respectively. ddH_2_O was used to prepare serial dilution of TPNT1 and was used as solvent control.

For yield reduction assay, the virus (multiplicity of infection, M.O.I. = 0.01) was pretreated with TPNT1 and was added to the Vero E6 cells (pretreat + infection), or TPNT1 was added during (infection) or after virus infection (post-infection). To determine the amount of SARS-CoV-2 virus RNA, RNA from the culture supernatants and cell extracts of infected cells were extracted, respectively, and was determined by qRT-PCR of E gene using the iTaq Universal Probes One-Step RT-PCR Kit (172-5140, Bio-Rad, USA) and the Applied Biosystems 7500 Real-Time PCR software (version 7500SDS v1.5.1). Plasmid containing partial E fragment was used as the standards to calculate the amount of viral load (copies/μL). Total cell lysates were prepared in lysis buffer (20 mM Tris, pH 7.5, 100 mM sodium chloride, 1% IGEPAL CA-630, 100 µM Na_3_VO_4_, 50 mM NaF, and 30 mM sodium pyrophosphate) for Western blotting. The primary antibodies used were anti-nucleoprotein (NP) of SARS-CoV-2 (40143-R019, Sino biological, 1:5s000) and anti-PCNA (Millipore Corporation, 1:5000). The infected cells from yield reduction assay were fixed, and then probed with a rabbit monoclonal antibody against the NP of SARS-CoV 2 (1:200; 40103-R019, Sinobiological, China) and FITC-labeled goat anti-rabbit IgG (1:300; Jackson ImmunoResearch, USA). The nuclei were stained with DAPI. All of the experiments involving SARS-CoV-2 virus were performed in the Biosafety Level-3 Laboratory of National Taiwan University College of Medicine.

### Cell toxicity assay

Vero E6 cells were seeded to the 96-well culture plate at a concentration of 2 × 10^4^ cells per well. Next day, each well was washed once with PBS before addition of DMEM containing 2% FBS and different concentrations of TPNT1. After 3 days of incubation at 37 °C, medium was removed and washed once with PBS. Then, buffer containing 0.1 M sodium acetate (pH = 5.0), 0.1% Triton X-100, and 5 mM p-nitrophenyl phosphate was added, and incubated at 37 °C for 30 min. After that, 1 N NaOH was added to stop the reaction. The absorbance was determined by ELISA reader at a wavelength of 405 nm. The percentage of cell viability was calculated using the following formula: cell viability % = [(At/As) × 100]%, where At and As refer to the absorbance of a tested concentration and cell control, respectively. The 50% cytotoxicity concentration (CC_50_) was defined as the concentration reducing 50% of cell viability.

### ACE2-Fc-biotin and spike binding by ELISA assay

The construction of ACE2-Fc-Biotin and the ELISA was described by our group previously^56^. Briefly, the 1-674 A.A. of the SARS-CoV-2 spike with humanized codons were PCR-amplified and fused with the Fc region of human IgG1 at its C-terminus as the tag. The soluble recombinant proteins generated by the Expi293F system (A14527, ThermoFisher Scientific, USA) were purified by Protein G Sepharose (Merck). The concentration and purity of recombinant proteins was determined by NanoDrop and polyacrylamide gel electrophoresis, respectively. The ELISA was established as described in our recently submitted manuscript. Briefly, 50 μL of 50 ng/mL purified 1-674 spike proteins were pre-coated onto the 96-well ELISA plate at 4 °C overnight. The plate was then washed three times with PBST (PBS containing 0.05% Tween-20) before blocking with blocking buffer (1% BSA, 0.05% NaN_3_ and 5% sucrose in PBS) at room temperature for 30 min. The plate was washed three times with PBST, and the mixture of serially diluted ACE2-Fc-Biotin with or without TPNT1 was added to the 96-well plate for 1 h at 37 °C. After that, the plate was washed three time with PBST and incubated with horseradish peroxidase (HRP)-conjugated streptavidin (1:500) at 37 °C for 30 min. After final wash with PBST, tetramethylbenzidine substrate (TMB) (T8665, Sigma) was added and incubated for 30 min before stopping the reaction by 50 µL of 1 N H_2_SO_4_. HRP activity was measured at 450 nm using ELISA plate reader (VERSAMAX).

### Syncytia formation

The syncytia formation assay was described by our group previously^56^. HEK293T cells were co-transfected with plasmid 5 µg pCR3.1-Spike and 0.5 µg pLKO AS2-GFP by lipofetamine 3000 (ThermoFisher, L3000015) before being used as effector cells (293 T-S) after 72 h. H1975 cells were transduced with lentivirus encoding full length ACE2 and were used as target cells (H1975-ACE2). Equal amounts of H1975 and H1975-ACE2 cells (2 × 10^5^ cells) were cultured in RPMI containing 10% FBS at 37 °C for 24 h. The 293 T-S cells were detached with 0.48 mM EDTA for 5 min, and the 293 T-S cells (1 × 10^5^) were incubated with H_2_O or TPNT1 at 37 °C for 1 h. After that, the mixture of TPNT1 and effector cells was added to target cells and incubated at 37 °C for another 24 h. The cells were fixed with 4% paraformaldehyde at room temperature for 30 min. The 293 T/Spike/EGFP cells fused or unfused with H1975-ACE2 cells were counted under an inverted fluorescence microscope (Leica DMI 6000B fluorescence microscope). The percent inhibition of syncytia formation was calculated using the following formula:

(100 − (H − L)/(E − L) × 100). H represents the total green fluorescent score in the individual picture. L represents the green fluorescent score in the negative control group with H1975-ACE2 replaced by H1975 as the target cells. E represents the green fluorescent score in each picture in TPNT1 group.

### Determination of anti-influenza activities

The reassortant viruses Influenza A/WSN/33 (H1N1), Influenza A/WSN/33 (H1N1) (NA H274Y), Influenza NIBRG14 (H5N1), Influenza NIBRG14 (H5N1) (NA H274Y) were created using 12-plasmid system that is based on cotransfection of mammalian cells with 8 plasmids encoding virion sense RNA under the control of a human PolI promoter and 4 plasmids encoding mRNA encoding the RNP complex (PB1, PB2, PA, and nucleoprotein gene products) under the control of a PolII promoter^57, 58^. Except hemagglutinin and neuraminidase genes, all other genes for production of recombinant viruses were gene Influenza A/WSN/33. Influenza A/WSN/33 (H1N1) and Influenza A/WSN/33 (H1N1) (NA H274Y) were produced by incorporation of the hemagglutinin and neuraminidase from A/WSN/33 with a mutation of H274Y on neuraminidase to create Influenza A/WSN/33 (H1N1) (NA H274Y). A/Viet Nam/1194/2004 (H5N1) (NA wt), Influenza NIBRG14 (H5N1) (NA H274Y) were produced by incorporation of the hemagglutinin and neuraminidase from A/Vietnam/1194/2004, with a mutation of H274Y on neuraminidase to create Influenza NIBRG14 (H5N1) (NA H274Y). The TCID_50_ (50% tissue culture infectious dose) was determined by incubation of serially diluted influenza virus in 100 μL solution with 100 μL of MDCK cells at 1 × 10^5^ cells/mL in 96-well microplates. The infected cells were incubated at 37 °C under 5% CO_2_ for 48–72 h and added to each well with 100 μL per well of CellTiter Aqueous Non-Radioactive Cell Proliferation Assay reagent (Promega). After incubation at 37 °C for 15 min, absorbance at 490 nm was read on a plate reader. Influenza virus TCID_50_ was determined using the Reed–Müench method^59, 60^. To test the anti-influenza activities of TPNT1, influenza virus at indicated titers was mixed with TPNT1 at various dilutions for 1 h at room temperature. The mixtures were used to infect MDCK cells at 1 × 10^5^ cells/mL in 96 wells. After 48 h of incubation at 37 °C under 5% CO_2_, the cytopathic effects were determined with CellTiter 96 AQueous Non-Radioactive Cell Proliferation Assay reagent (Promega). The experiments involving influenza viruses were performed in the Biosafety Level-2 Laboratory of Genomics Research Center, Academia Sinica.

### Statistical analysis

Data were expressed as the mean ± standard deviation (SD). A two-tailed Student’s t-test was used for the comparison of continuous variables, and a *P* < 0.05 was considered statistically significant (*P* < 0.05*; *P* < 0.01**; *P* < 0.001***).

## Supplementary Information

Supplementary Information

## Data Availability

This manuscript has been deposited in the Research Square preprint platform and the pre-print link is https://www.researchsquare.com/article/rs-52066/v1. All methods were carried out in accordance with relevant guidelines and regulations.
