# Supplementary material for: Nanoparticle composite TPNT1 is effective against SARS-CoV-2 and influenza viruses
Source: Sci Rep. 2021 Apr 22;11:8692. doi: 10.1038/s41598-021-87254-3 (PMC8062499; doi:10.1038/s41598-021-87254-3)
Supplement: Supplementary file 1 — Supplementary Information [file 41598_2021_87254_MOESM1_ESM.docx]

**Supporting Information**

**Nanoparticle composite TPNT1 is effective against SARS-CoV-2 and influenza viruses**

Sui-Yuan Chang ^a, b^, Kuo-Yen Huang ^c^, Tai-Ling Chao ^a^, Han-Chieh Kao ^a^, Yu-Hao Pang ^a^, Lin Lu ^d^, Chun-Lun Chiu ^d^, Hsin-Chang Huang ^d^, Ting-Jen Rachel Cheng ^e^, Jim-Min Fang ^e, f,^ *, Pan-Chyr Yang ^c, g,^ *

^a^ Department of Clinical Laboratory Sciences and Medical Biotechnology, National Taiwan University College of Medicine, No. 1, Sec. 1, Ren-Ai Rd., Taipei 10002, Taiwan.

^b^ Department of Laboratory Medicine, National Taiwan University Hospital, No. 7, Chung-Shan South Rd., Taipei 10002, Taiwan.

^c^ Institute of Biomedical Sciences, Academia Sinica, No. 128, Sec. 2, Academia Rd., Taipei 11529, Taiwan.

^d^ Tripod Nano Technology, No. 171, Sec. 1, Mei Shi Rd., Yang Mei District, Taoyuan 32656, Taiwan.

^e^ The Genomics Research Center, Academia Sinica, No. 128, Sec. 2, Academia Rd., Taipei 11529, Taiwan.

^f^ Department of Chemistry, National Taiwan University, No. 1, Sec. 4, Roosevelt Rd., Taipei 10607, Taiwan.

^g^ Department of Internal Medicine, National Taiwan University Hospital and National Taiwan University College of Medicine, No. 7, Chung-Shan South Rd., Taipei 10002, Taiwan.

* Corresponding authors:

*E-mail addresses*: [jmfang@ntu.edu.tw](mailto:jmfang@ntu.edu.tw) (J.-M. Fang), [pcyang@ntu.edu.tw](mailto:pcyang@ntu.edu.tw) (P.-C. Yang)

| **Contents** | **Pages** |
| --- | --- |
| Figure S1. Inhibition of the viral nucleocapsid (NP) protein in the presence of TPNT1. | S2-S3 |
| Figure S2. Inhibition of the viral nucleocapsid (NP) protein by adding TPNT1 at the pretreat + infection, infection-only, and post-infection stages. | S4 |

**a.**

**
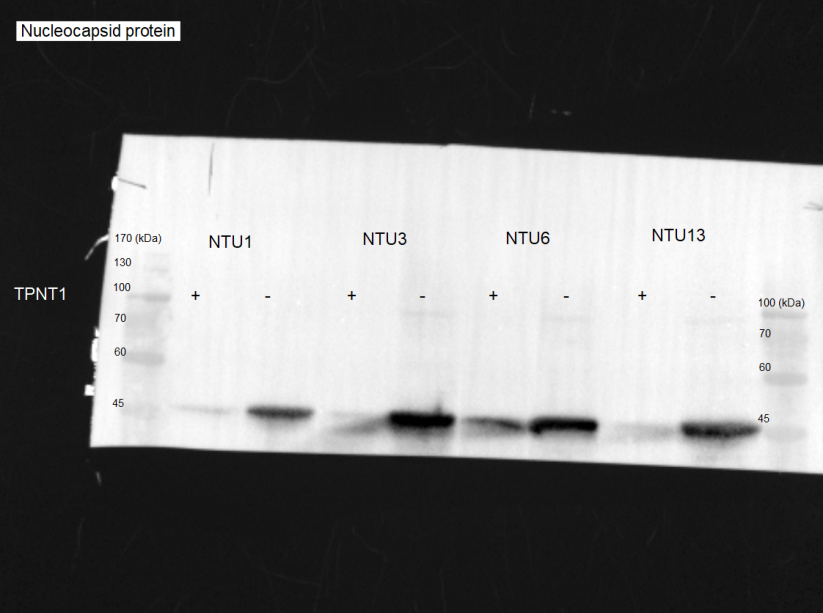
**

**b.**

**
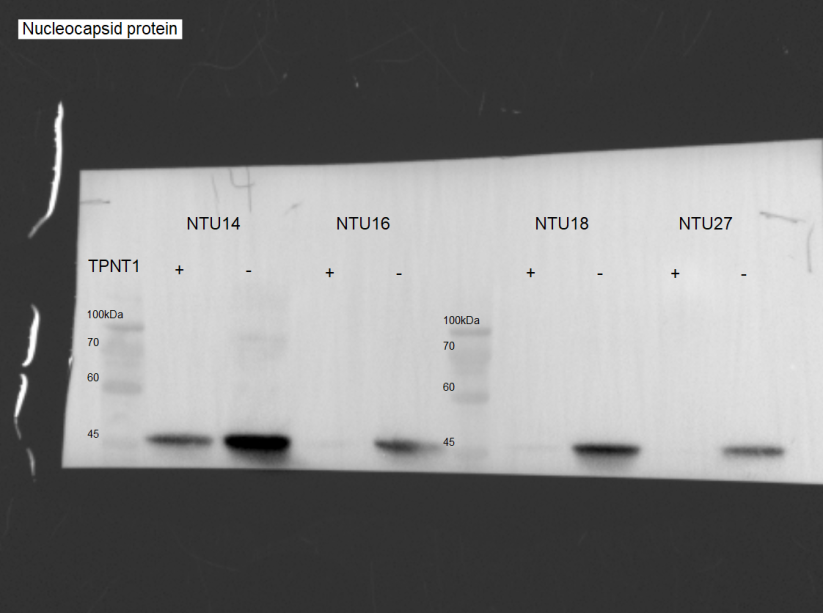
**

**c.**

**
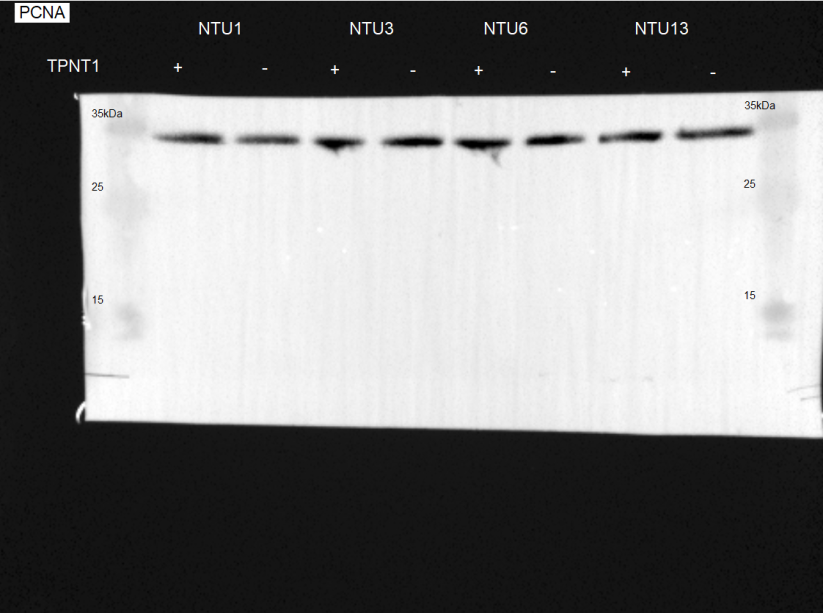
**

**d.**

**
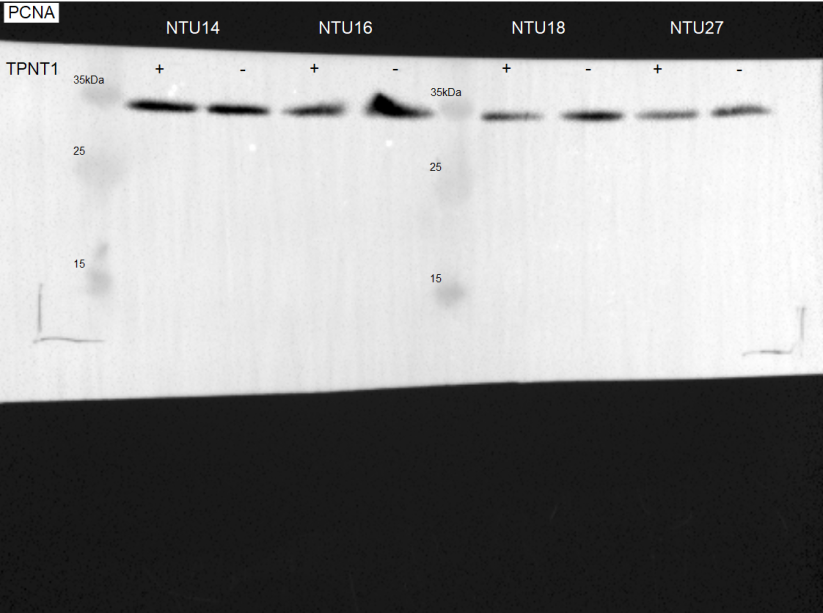
**

**Figure S1. Inhibition of the viral nucleocapsid (NP) protein in the presence of TPNT1.** Full length Western blot of (**a-b**) the viral nucleocapsid (NP) proteins and (**c-d**) the PCNA control was shown. (**a, c**) represent cell lysates from NTU1, 3, 6, and 13 infected cells in the presence (+) or absence (-) of TPNT1; (**b, d**) represent cell lysates from NTU14, 16, 18, and 27 infected cells in the presence (+) or absence (-) of TPNT1. After transferring of proteins from the 10% SDS-PAGE gels to polyvinylidene diﬂuoride (PVDF) membranes, the membranes were cut into half for subsequent detection of NP and PCNA proteins, respectively.

**a.**

**
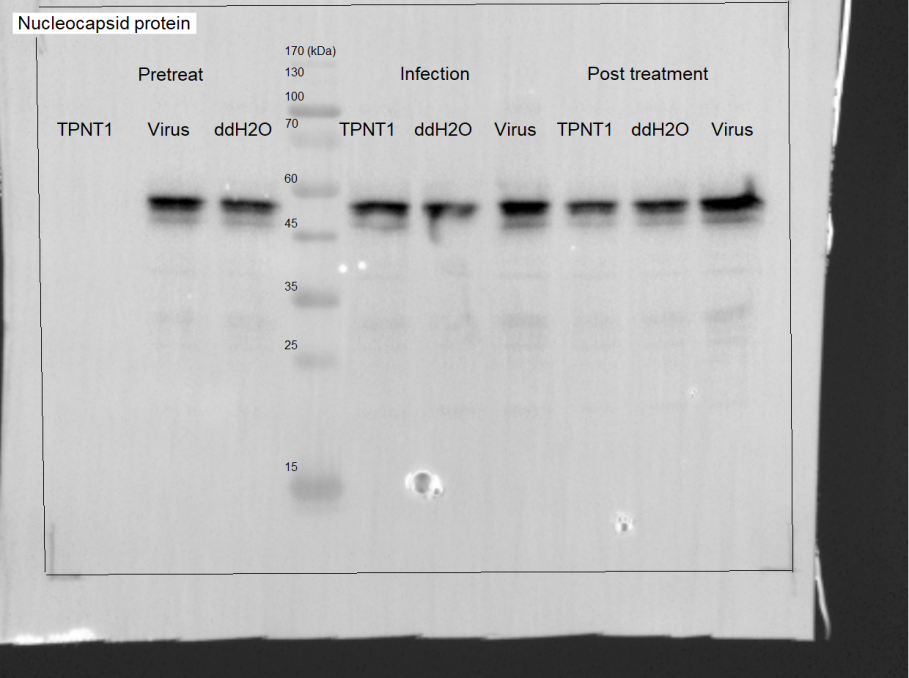
**

**b.**

**
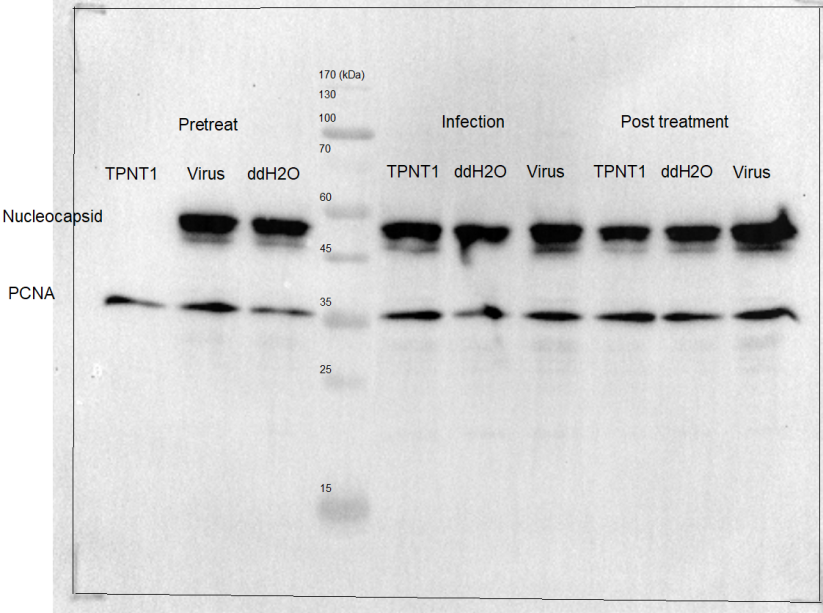
**

**Figure S2.** **Inhibition of the viral nucleocapsid (NP) protein by adding TPNT1 at the pretreat + infection, infection-only, and post-infection stages.** Full length Western blot of (**a**) the viral nucleocapsid (NP) proteins and (**b**) the PCNA control. After electro-transferring, the polyvinylidene diﬂuoride (PVDF) membrane was used to detect the presence of NP proteins first, then probing with the anti-PCNA antibody.
